# Comparison of content and psychometric properties for assessment tools used for brain tumor patients: a scoping review

**DOI:** 10.1186/s12955-021-01863-0

**Published:** 2021-10-09

**Authors:** Lelde Ģiga, Anete Pētersone, Silva Čakstiņa, Guna Bērziņa

**Affiliations:** 1 Riga Stradiņš University, Riga, Latvia; 2 Riga East University Hospital, Riga, Latvia; 3 Department of Rehabilitation, Riga Stradiņš University, Anniņmuižas Boulevard 26a, Riga, 1067 Latvia

**Keywords:** International Classification of Functioning, Disability and Health (ICF), Linking, Psychometric properties, Outcome measures, Brain Tumor

## Abstract

**Aims:**

To determine the most frequently utilized functional status assessment instruments for patients with brain tumors, compare their contents, using the International Classification of Functioning, Disability and Health (ICF), and their psychometric properties.

**Methods:**

A scoping review was conducted to explore possible assessment instruments and summarize the evidence. A systematic literature search was performed for identification of the frequently used functional assessment tool in clinical trials in PubMed, ScienceDirect, and ProQuest databases. The content of most used instruments was linked to the ICF categories. The psychometric qualities of these assessment tools were systematically searched and analyzed.

**Results:**

Nine most used assessment tools in clinical trials were identified. The most frequently used assessment instrument is the Karnofsky Performance Scale, which is developed for a general assessment of oncological patients. Out of four self-assessment tools, two were disease-specific (EORTC QLQ-BN20 and FACT-Br), EORTC QLQ-C30 has been shown good psychometric properties in patients with brain tumors as well as in patients with various oncological diseases, similar to the SF-36, it is used in patients with brain tumors as well as in patients with various diseases. The Functional Independence Measure and the Barthel Index were two objective assessment tools that described functioning, but two were neuropsychological tests (MMSE and Trial Making Test). Two hundred eighty-three meaningful concepts were identified and linked to 102 most relevant second-level categories covering all components of the ICF. Forty-nine studies reporting psychometric properties of those nine assessment tools were identified, indicating good reliability and validity for all the instruments.

**Conclusion:**

Nine most frequently utilized functional status assessment instruments for patients with brain tumors represent all components of the ICF and have good psychometric properties. However, the choice of the tool depends on the clinical question posed and the aim of its use.

**Supplementary Information:**

The online version contains supplementary material available at 10.1186/s12955-021-01863-0.

## Introduction

Based on 2015 statistics, patients with brain tumors make up a total of 5% of all oncology patients in Latvia [1]. As the medical industry, diagnostic capabilities, and technologies for treating primary tumors evolve, the survival rates for individuals diagnosed with primary brain tumors have increased significantly [1, 2]. Tumor localization, anatomical distribution, and volume are determinants before and after primary treatment. The most common symptoms for brain tumors usually include headache, nausea, vomiting, partial and generalized seizures, cognitive impairment, and ataxia. These symptoms may also arise from common treatment strategies used for brain tumor patients such as chemotherapy, radiation therapy, and surgery. It is estimated that 75% of all patients with brain tumors show symptoms of focal neurological deficiency [3], which greatly affects one’s level of functioning, as well as the quality of life.

Numerous articles discuss the role of rehabilitation in tumor cases, while others discuss the positive effects of rehabilitation for patients with brain tumors compared to patients with stroke or after a traumatic brain injury [4, 5]. All of these articles demonstrate positive outcomes in restoring functioning [6, 7]. Bartolo M. et al. have demonstrated that rehabilitation is very effective if initiated as early as possible after primary treatment for brain tumor patients [5].

To assess the rehabilitation needs and outcomes for this population, a specific functional disability assessment tool is necessary [8]. The use of appropriate assessment tools could improve rehabilitation planning that in turn would lead to better outcomes, including patients’ quality of life. Currently, no standardized protocols are provided for evaluation of persons with brain tumors. The International Classification of Functioning, Disability and Health (ICF) provides a framework for coding large-scale health information, a common standardized language for identifying and comparing functional assessment tools, and provides valuable information to develop an evidence-based standardized evaluation protocol for patients with brain tumor [9].

The aim of this study was to determine the most frequently utilized functional assessment instruments for patients with brain tumors, compare their contents, using the International Classification of Functioning, Disability and Health, and analyze their psychometric properties.

## Methods

### Identification of assessment tools

**Fig. 1:**
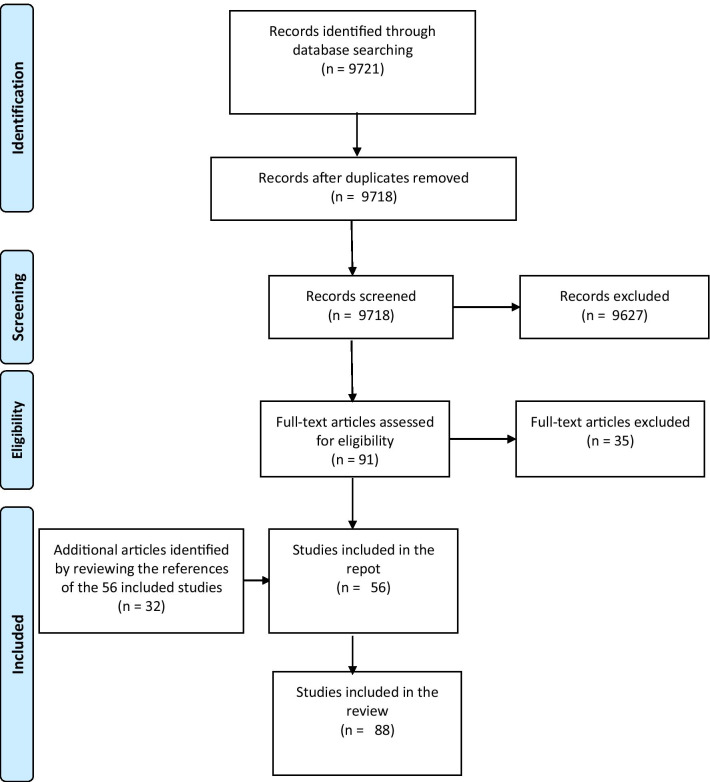
Flow diagram of the systematic literature search

A scoping review was conducted according to Joanna Briggs institute guidelines [10]. PubMed, ScienceDirect, and ProQuest databases were searched (last updated August 2020) for publications since 2000 using the following keywords: “brain neoplasm” or “meningioma” or “glioblastoma” or “intracranial neoplasm” or “brain cancer” or “outcome assessment” or “treatment outcome” or functional outcome mentioned in the title/abstract. Studies referring in the title or abstract to assessment tools used to assess people diagnosed with brain tumors older than 18 years were included. Original research studies randomized controlled clinical trials, observational studies, cross-sectional studies, qualitative studies were included in which authors reported using a tool to assess functioning in persons with brain tumors. Studies were included even if the tool was not initially designed to assess functioning. Studies were excluded if they addressed genetic, laboratory, and animal research. Systematic reviews, secondary analyses of published data, validity studies, protocols, letters, were also excluded from this report. All searches were limited to journal articles written in English; the search results were compiled in the reference management system EndNote where duplicates were removed. A summary of the search procedure is shown in Fig. 1.

Data collection was based on the Joanna Briggs Institute Manual for Evidence Synthesis Chapter 11.2 Development of a Scoping Review protocol [10]. General study data (year of publication, country, study design), available data on participants (number, diagnosis), and assessment tools used in the study were recorded. Assessment tools that were used in more than 9% of all studies the scoping review using frequency analysis. The choice of this cut-off point was based on the distribution of frequencies as well as on substantive considerations of the list of instruments.

### Linking to the ICF

All assessment instruments identified in the study meeting selection criteria were classified using the ICF linking guidelines. The ICF linking guidelines state that before starting the process of linking health-status measures to the ICF categories, identification of all meaningful concepts within each item of the health status measure needs to be performed. According to the rules, the interval of time cannot be linked to the ICF, also, if a meaningful concept of an item is explained by examples, both the concept and the examples are to be linked, while technical measures can be linked by defining the purpose and then linking it with the ICF category [11, 12]. Two independent medical professionals (authors LG and SS) separately identified the meaningful concepts within the analyzed instruments and linked them to the ICF concepts. The raters met and discussed any discrepancies to achieve a consensus classification for the instruments and GB served as a third rater, in case the consensus could not be reached. Identified categories within each of the analyzed instruments were organized according to the structure of the ICF. Further, the content of the instruments was compared to identify categories that overlap between the instruments and those that are unique for specific tools. The perspective adopted in health information and categorization of response for self-assessment tools were also reported [11].

### Psychometric properties

Following the search methodology developed by PubMed [13], the electronic database MEDLINE (PubMed) was searched for studies that reflect the psychometric properties of a particular assessment tool. First, a search was performed using a diagnosis-specific MeSH terms and key words identified in the search methodology and the names of assessment tools. Headline screening identified studies that reflected one of the psychometric properties of a given instrument (reliability: internal coherence; test/retest method, evaluator reliability. Validity: content validity; criterion validity; construct validity) specific to patients with brain tumors. If psychometric properties for chosen assessment tools were not identified, the search was repeated excluding diagnosis-specific MeSH terms, thus conducting a search for studies covering different diagnoses. Headline screening then identified studies that reflected one of the psychometric properties of a given instrument for various diagnoses. The interpretation of the psychometric properties is given in Table 1.

**Table 1:** The interpretation of the psychometric properties

**Reliability**		
Internal reliability	+	Cronbach’s ɑ or ICC ≥ 0.70
	**−**	Cronbach’s ɑ or ICC < 0.70
Test/retest method	+	ICC ≥ 0.70 or Pearson correlation coefficient/ Spearman rank correlation coefficient ≥ 0.80
	**-**	ICC < 0.70 or Pearson correlation coefficient/Spearman rank correlation coefficient < 0.80
Interrater reliability	+	ICC ≥ 0.70 or Pearson correlation coefficient/ Spearman rank correlation coefficient ≥ 0.80
	**−**	ICC < 0.70 or Pearson correlation coefficient/Spearman rank correlation coefficient < 0.80
**Validity**		
Content validity	+	The content of the assessment instrument is adequate, comprehensive, questions and tasks chosen to adequately reflect the content to be evaluated
	**-**	Not all selected questions and tasks reflect the content, content is not relevant, comprehensive
Criterion validity	+	Significant and stable relation between measurement and another instrument (r ≥ 0.70) or with start/end measurement
	**−**	Poor measurement correlation with another instrument (r < 0.70) or start / end measurement
Structural validity	+	Correlation with instruments measuring the same ≥ 0.50 or correlation higher for unrelated elements in the instrument than for unrelated ones
	**-**	Correlation with instruments measuring the same < 0.50 or correlation with related elements in the instrument is lower than unrelated ones
**Responsiveness**		
Responsiveness	+	Able to detect clinically significant changes over time
	**−**	Cannot detect clinically significant changes over time

Cronbach’s ɑ, Chronbach's ɑ coefficient; ICC, interclass correlation coefficient

## Results

### Identification of assessment tools

The initial search strategy returned 9721 articles. The duplicates were removed, titles and summaries were revised, following the exclusion of articles that did not meet the selection criteria, in result 56 articles were included in the scoping review.

To make the search as comprehensive as possible, references from the 56 included articles were studied and an additional 32 articles were included after applying the selection criteria.

As a result, a total of 88 studies were included in the report; 31 were administered in the United States, 42 in Europe (8 in Italy, 8 in the Netherlands, 6 in Norway, 4 in France, Germany and England each 3, Austria, Turkey, Sweden 2 studies each, Poland, Switzerland, Denmark, and Finland each 1), 2 in Australia, 7 in Canada, and 4 in Asian countries (Korea, Israel, Iran). The studies look at groups of patients with various brain tumor diagnoses. The 74 articles included patients with primary tumors, of which 26 were diagnosed with glioma, 3- oligodendroglioma, 1- oligoastrocytoma, 3- astrocytoma, 4- adenoma, 1- meningioma, 1 case study had a mixed group with patients suffering from meningioma and glioblastomas. 28 of the studies did not categorize patients by their histologic type; instead, patients with primary brain tumors were evaluated. 9 studies evaluated patients with secondary brain tumors or with brain metastases. In 4 of the included studies, the functional abilities of patients with brain tumors are compared to those of a stroke patient or a patient with a brain injury.

All instruments mentioned in the articles were identified, yielding 86 assessment tools which are summarized in Additional file 1. According to research methodology, 9 assessment tools that were used in more than 9% of the research articles included in the study were used for further analysis: A list of these instruments, their abbreviations, and the number of articles that have used that instrument are summarized in Table 2. Out of nine instruments included in the study,

**Table 2:** The most frequently used assessment instruments

Assessment instrument	Abbreviation	N of studies mentioned	Frequency (%)
Karnofsky Performance Scale	KPS	42	48
Mini-Mental State Examination	MMSE	20	23
EORTC Quality of Life Questionnaire-Core 30	EORTC QLQ-C30	18	20
EORTC Quality of Life Questionnaire-Brain Neoplasm 20	EORTC QLQ-BN20	15	17
Functional Independence Measure	FIM	13	15
Trail Making Test	TMT	13	15
Barthel Index	BI	9	10
Functional Assessment of Cancer Therapy-Brain	FACT-BR	8	9
36-Item Short Form Health Survey	SF-36	8	9

two are specific for patients with brain tumors: ORTC Quality of Life Questionnaire-Brain Neoplasm 20 (EORTC QLQ-BN20), Functional Assessment of Cancer Therapy-Brain (FACT-Br), one is specific for patients with oncological diseases—Karnofsky Performance Scale (KPS), four: Mini-mental State Examination (MMSE), Functional Independence Measure (FIM), Trial Making Test (TMT), Barthel Index (BI) and 36-Item Short Form Health Survey (SF-36)) are used for patients with various diagnoses. The EORTC Quality of Life Questionnaire 30 (EORTC QLQ-C30) has been shown to be valid, reliable, and responsive in patients with brain tumors as well as in patients with various oncological diseases. Similar, to the 36-item Short Form Health Survey (SF-36), it is used in patients with brain tumors as well as in patients with various diseases.

### Linking to the ICF

In total, 283 meaningful concepts were identified within all nine assessment instruments and linked to 394 most precise categories of the ICF. The detailed description of the linking is shown in Table 3. In two cases, the meaningful concepts could be linked most precisely to the component of Activities and Participation. In 12 cases, it was the first level or chapter under the component of Activities and Participation. The content of the assessment tools was linked to 102 most relevant second-level categories of the ICF in total. Thirty-four of these categories were under the component of Body Functions and Structures, 50 – under Activities and Participation, and 18 – Environmental Factors. Detailed comparison of the content between assessment tools are shown in Tables 4, 5, and 6 for components of Body Functions and Structures, Activities and Participation and Environmental Factors, respectively. No appropriate ICF category was found for 54 items following the ICF linking guidelines.

**Table 3:** Summary of linking the nine most frequently used assessment tools to the ICF

	KPS	MMSE	EORTC QLQ-C30	EORTC QLQ-BN20	FIM	TMT	BI	FACT-BR	SF-36
N of meaningful concepts	32	11	42	22	19	NA	32	63	62
N of categories identified	71	18	52	29	31	16	39	66	72
N of unique categories identified	22	15	39	19	29	16	22	52	27
Perspective	Desc	Desc	Appr	Appr	Dep	Desc	Dep	Appr	Appr
Categorization			Int	Int				Int	Int
*Body functions*									
2nd level		4	9	7	3	6	2	16	4
3d and 4th level		10	6	6		1		5	
*Body structures*									
2nd level								2	
*Activities and participation*	1								1
1st level	1		5					2	4
2nd level	4		15	5	20	9	8	17	6
3d level		1	4	1	3		5	1	12
*Environmental factors*									
2nd level	13	9			1		7		
3d level	3								
*Not classified*									
Nc-health condition	6			2				2	
Nc-Quality of life			1	2				13	5
Nd-general health			1					2	6
Nd-physical health			3					1	2
Nd-mental health									2
Nd-disability	2								
Personal factors	1							1	2

Desc, descriptive; Dep, dependency; Appr, appraisal; Int, intensity; NC, not covered; ND, not definable

**Table 4:** Content comparison of assessment tools linked to the component of Body Functions and Structures of the ICF

		KPS	MMSE	EORTC QLQ-C30	EORTC QLQ-BN20	FIM	TMT	BI	FACT-BR	SF-36	Total
*Body functions*											
**b1**	**Mental functions**										
b110	Consciousness functions				x						1
b114	Orientation functions		xxx								1
b117	Intellectual functions						x				1
b126	Temperament and personality functions			x					xxx		2
b130	Energy and drive functions			xxx					x	x	3
b134	Sleep functions			x					x		2
b140	Attention functions		x	x		x	x		x		5
b144	Memory functions		x	x		x			x		4
b152	Emotional functions			xxx						xxx	2
b156	Perceptual functions		x		x		x		x		4
b160	Thought functions		x				x		x		3
b164	High level cognitive functions						x				1
b167	Mental functions of language		xxx		x				x		3
b172	Calculation functions		x								1
b176	Mental functions of sequencing complex movements		x								1
**b2**	**Sensory functions and pain**										
b210	Seeing functions				xxx		x				2
b260	Proprioceptive function				x				x		2
b265	Touch function								xxx		1
b270	Sensory functions related to temperature and other stimuli								x		1
b280	Sensation of pain			x	x				x	x	4
**b4**	**Functions of the cardiovascular, haematological, immunological and respiratory systems**										
b440	Respiration functions			x							1
b455	Exercise tolerance functions			xxx							1
**b5**	**Functions of digestive, metabolic and endocrine systems**										
b510	Ingestion functions			x							1
b525	Defecation functions			x		x		x			3
b535	Sensations associated with the digestive system			x					x		2
**b6**	**Genitourinary and reproductive functions**										
b620	Urination functions				x	x		x			3
**b7**	**Neuromusculosceletal and movement related functions**										
b730	Muscle power functions			x	xxx				x	x	4
b740	Muscle endurance functions				x						1
b760	Control of voluntary movement functions				x				x		2
b780	Sensations related to muscles and movement functions								x		1
**b8**	**Functions of the skin and related structures**										
b840	Sensation related to the skin				x				x		2
b850	Functions of hair				x						1
Total		0	8	13	12	4	6	2	17	4	
*Body structures*											
s730	Structure of upper extremity								x		1
s750	Structure of lower extremity								x		1
Total		0	0	0	0	0	0	0	2	0	

x = 1 or 2 items included, xxx = 3 or more items included

**Table 5:** Content comparison of assessment tools linked to the component of Activities and Participation of the ICF

		KPS	MMSE	EORTC QLQ-C30	EORTC QLQ-BN20	FIM	TMT	BI	FACT-BR	SF-36	Total
**d1**	**Learning and applying knowledge**									xxx	1
d110	Watching			x			x				2
d115	Listening						x				1
d160	Focusing attention						x				1
d163	Thinking						x				1
d166	Reading			x	x		x		x		4
d175	Solving problems					x			x		2
d177	Making decisions								x		1
**d2**	**General tasks and demands**										
d220	Undertaking multiple tasks						x				1
d230	Carrying out daily routine			x						x	2
d240	Handling stress and other psychological demands								x		1
**d3**	**Communication**								x		1
d310	Communicating with receiving spoken messages					x					1
d315	Communicating with receiving nonverbal messages					x	x				2
d320	Communicating with—receiving—formal sign language messages					x					1
d325	Communicating with—receiving—written messages					x					1
d330	Speaking				x	x			x		3
d335	Producing nonverbal messages		x			x					2
d340	Lifting and carrying objects			x		x					2
d345	Writing messages					x			x		2
d350	Conversation				x						1
**d4**	**Mobility**			x							1
d410	Changing basic body position							x		x	2
d415	Maintaining a body position			x	x				x		3
d420	Transferring oneself					xxx		x			2
d430	Lifting and carrying objects									xxx	1
d440	Fine hand use						x				1
d455	Hand and arm use						x				1
d450	Walking			x	x	x		xxx		xxx	5
d455	Moving around					x		xxx		xxx	3
d460	Moving around in different locations			x							1
d465	Moving around using equipment					x		x			2
d475	Driving								x		1
**d5**	**Self-care**	xxx							x		2
d510	Washing oneself			x		x		x		x	4
d520	Caring for body parts					x		xxx	x		3
d530	Toileting			x		x		x			3
d540	Dressing			x		x		x	x	x	5
d550	Eating			x		x		x	x		4
d560	Drinking								x		1
d598	Self-care										0
**d6**	**Domestic life**			x						x	2
d620	Acquisition of goods and services					x					1
**d7**	**Interpersonal interactions and relationships**			x						x	2
d710	Basic interpersonal interactions					x					1
d720	Complex interpersonal interactions					x					1
d750	Informal social relationships									xxx	1
d760	Family relationships			x	x				x	x	4
d770	Intimate relationships								x		1
**d8**	**Major life areas**			x						x	2
d840	Apprenticeship (work preparation)	xxx		x							2
d845	Acquiring, keeping and terminating a job	xxx		x							2
d850	Remunerative employment	xxx		x					x	x	4
d855	Non-remunerative employment	xxx		x							2
d870	Economic self-sufficiency			x							1
**d9**	**Community, social and civic life**			x						x	2
d910	Community life								x		1
d920	Recreation and leisure			x					x	xxx	3
Total		6	1	23	6	21	9	10	19	16	

x = 1 or 2 items included, xxx = 3 or more items included

**Table 6:** Content comparison of assessment tools linked to the component of Environmental Factors of the ICF

		KPS	MMSE	EORTC QLQ-C30	EORTC QLQ-BN20	FIM	TMT	BI	FACT_BR	SF-36	Total
**e1**	**Products and technology**										
e110	Products or substances for personal consumption	x									1
e115	Products and technology for personal use in daily living	x				x		x			3
e120	Products and technology for personal indoor and outdoor mobility and transportation	xxx						x			2
**e3**	**Support and relationships**										
e310	Immediate family	xxx						x	x		3
e315	Extended family	xxx						x	x		3
e320	Friends	xxx						x	x		3
e325	Acquaintances, peers, colleagues, neighbors and community members	x									1
e330	People in positions of authority	x									1
e335	People in subordinate positions	x									1
e340	Personal care providers and personal assistants	xxx						x	x		3
e345	Strangers	x									1
e350	Domesticated animals	x									1
e355	Health professionals	x						x	x		3
**e4**	**Attitudes**										
e410	Individual attitudes of immediate family members								x		1
e415	Individual attitudes of extended family members								x		1
e420	Individual attitude of friends								x		1
**e5**	**Services, systems and policies**										
e575	General social support services, systems and policies	x									1
e580	Health services, systems and policies	xxx									1
Total		15	0	0	0	1	0	7	8	0	

x = 1 or 2 items included, xxx = 3 or more items included

Evaluating all 9 assessment tools, the most related ICF categories in the body function domain were b140 attention functions (n = 5), b144 memory functions (n = 4), b156 perceptual functions (n = 4), b280 sensation of pain (n = 4) and b730 muscle power functions (n = 4). FACT-BR, BN-20 and MMSE contained most concepts related to the Body functions and Structures. Five out of nine analyzed assessment tools included concepts on d450 walking and d540 dressing. The BN-20 questionnaire contained meaningful concepts that could be linked to 23 s level categories of the Activities and Participation, covering all domains of this component. FIM was linked to 21 categories that did not cover chapters of Major life areas and Community, social and civic life.

### Psychometric properties

For psychometric properties that are specific for brain tumor diagnosis, search in PubMed yielded 578 results for KPS, 18 for MMSE, 55 for EORTC QLQ-C30, 6 for EORTC QLQ-BN20, 5 for FIM, 36 for TMT, 14 for BI, 21 for FACT-Br, and 4 for SF-36. Headline screening resulted in identifying 1 study for EORTC QLQ-C30, 4 studies for EORTC QLQ-BN20, 3 studies for FACT- Br, and 1 study for SF-36. A search strategy for various diagnoses was implemented for the remaining assessment instruments as well as SF-36 and EORTC QLQ-C30 due to the previous search strategy yielding only 1 result. As a result, for further analysis, 4 articles for KPS, 5 for MMSE, 1 + 5 for EORTC QLQ-C30, 4 for EORTC QLQ-BN20, 10 for FIM, 2 for TMT, 7 for BI 3 for FACT-Br, and 8 for SF-36 were included in this review. The psychometric properties for assessment instruments EORTC QLQ-C30, EORTC QLQ-BN20, FACT-Br, and SF-36, that are specific to brain tumor diagnosis are summarized in Table 7. The psychometric properties analyzed in mixed diagnosis studies for EORTC QLQ-C30, MMSE, SF-36, BI, FIM, KPS, TMT are summarized below, see Table 8.

**Table 7:** The psychometric properties of assessment tools used in patients with a diagnosis of brain tumor

Assessment instrument	Reliability				Validity			Responsiveness	Time	Research group	References
	Test–retest	Inter-rater	Intra-rater	Content		Structural	Criterion				
EORTC QLQ-BN20			+			+		+		n = 891, brain tumor	Taphoornet al. [27]
			+*			+			< 10 min	n = 350, glioma, meningioma	Shin and Kim [28]
			+			+		+		n = 194, brain tumor	Khoshnevisan et al. [29]
			+			+				n = 100, brain tumor	Bunevičius et al. [30]
EORTC QLQ-C30			+*				−	+	13–14 (SD 12) min	n = 366, brain tumor	Cheng et al. [31]
FACT-Br	+		+			+				n = 500, brain tumor	Arli and Gurkan [32]
	+^§^		+^†,‡^			+			5 to 10 min	n = 101, brain tumor	Weitzner et al. [33]
	+^†^					+		+		n = 62, metastases in the brain	Thavarajah et al. [34]
SF-36			+			+			10 [40–45] min	n = 277, brain tumor	Bunevičius et al. [30]

For interpretation of “+” and “−” for each property: Internal reliability: “+” Cronbach’s ɑ or ICC ≥ 0.70; “−” Cronbach’s ɑ or ICC < 0.70;

Test/retest method: “+” ICC ≥ 0.70 or Pearson correlation coefficient/ Spearman rank correlation coefficient ≥ 0.80; “−” ICC < 0.70 or Pearson correlation coefficient/Spearman rank correlation coefficient < 0.80;

Interrater reliability: “+” ICC ≥ 0.70 or Pearson correlation coefficient/ Spearman rank correlation coefficient ≥ 0.80; “−” ICC < 0.70 or Pearson correlation coefficient/Spearman rank correlation coefficient < 0.80;

Content validity: “+” The content of the assessment instrument is adequate, comprehensive, questions and tasks chosen to adequately reflect the content to be evaluated; “ –” Not all selected questions and tasks reflect the content, content is not relevant, comprehensive;

Criterion validity: “+” Significant and stable relation between measurement and another instrument (r ≥ 0.70) or with start/end measurement; “ –” Poor measurement correlation with another instrument (r < 0.70) or start/end measurement;

Structural validity: “+” Correlation with instruments measuring the same ≥ 0.50 or correlation higher for unrelated elements in the instrument than for unrelated ones; “−” Correlation with instruments measuring the same < 0.50 or correlation with related elements in the instrument is lower than unrelated ones;

Responsiveness: “+” Able to detect clinically significant changes over time; “−” Cannot detect clinically significant changes over time

SD, standard deviation

^*^Except Cognitive scale; ^†^Except Social, family wellbeing domains; ^‡^Except Interaction with a physician domain; ^§^Functional Assessment of Cancer Therapy—General questionnaire analyzed

**Table 8:** The psychometric properties analyzed in mixed diagnosis studies

Assessment instrument	Reliability			Validity				Responsiveness	Time	Research group	References
	Test–retest	Inter-rater	Intra-rater		Content	Structural	Criterion				
EORTC QLQ- C30			+*			+				n = 89, myeloma	Kontodimopoulos et al. [35]
			+*			+				n = 105, breast cancer	Kontodimopoulos et al. [35]
			+*			+				n = 28, lung cancer	Ozturk et al. [36]
			+*			+	+			n112, lung cancer	Nicklasson and Bergman [37]
			+			+			11 min	n = 305, lung cancer	Aaronson et al. [38]
FIM			+			+				n = 93,829, rehabilitation patients	Stineman et al. [39]
		+	+			+				n = 62, SCI; n = 51, stroke	Küçükdeveci et al. [40]
	+					+				n = 49, age > 50	Pollak et al. [41]
		+								n = 50, SCI	Karamehmetoglu et al. [42]
		+								n = 11 studies	Ottenbacher et al. [43]
			+			+		+		n = 11 102, mixed diagnosis	Dodds et al. [44]
						+	+			n = 1502, neurologic deficit	Ng et al. [45]
						+				n = 102, acute stroke	Tur et al. [46]
						+				n = 48, brain trauma	Hall et al. [47]
								+	30–40 min	n = 516, mixed diagnosis	Coster et al. [48]
BI	+	+	+			+				n = 459, stroke	Oveisgharan et al. [49]
			+			+				n = 258, stroke	Shah et al. [50]
		+			+			+	10.1 min (± 1.56)	n = 50, neurologic deficit	Rödén-Jüllig et al. [51]
			+			+				n = 90, multiple sclerosis	Nicholl et al. [52]
		+								n = 273, neurologic deficit	Rollnik [53]
							+			n = 75, brain trauma	Liu et al. [54]
								+		n = 259, vascular brain damage; n = 107 brain trauma	Houlden et al. [55]
SF-36	+		+			+				n = 1980, mixed	Brazier et al. [56]
			+			+				n = 124, stroke	Anderson et al. [57]
			+			+				n = 252, n-198, endometriosis	Stull et al. [58]
	+		+							n = 37, Parkinsons	Steffen and Seney [59]
	+									n = 209, stroke	Dorman et al. [60]
			+							n = 514, brain trauma	Guilfoyle et al. [61]
							+			n = 120, brain trauma	Paniak et al. [62]
MMSE			+			+		+		n = 207, brain trauma	Elhan et al. [63]
	+					+			10 min	n = 63, no impairments; n = 206, cognitive impairments	Folstein et al. [64]
		+								n = 48, age > 50	Molloy et al. [65]
			+							n = 72, stroke	Toglia et al. [66]
								−		n = 112, stroke	Blake et al. [67]
KPS		+				+				n = 52, tumor	Yates et al. [68]
		+					+			n = 100, mixed diagnosis	Grieco et al. [69]
		+					+			n = 47, tumor	Mor et al. [70]
		+					+			n = 93, tumor	Schag et al. [71]
TMT	+					+			A part 180 s B part 300 s	n = 484, age > 50	Cangoz et al. [71]
					+	+				n = 117, neurologic deficit	O’Donnell et al. [72]

For interpretation of “+” and “−” for each property: Internal reliability: “+” Cronbach’s ɑ or ICC ≥ 0.70; “−” Cronbach’s ɑ or ICC < 0.70;

Test/retest method: “+” ICC ≥ 0.70 or Pearson correlation coefficient/ Spearman rank correlation coefficient ≥ 0.80; “−” ICC < 0.70 or Pearson correlation coefficient/Spearman rank correlation coefficient < 0.80;

Interrater reliability: “+” ICC ≥ 0.70 or Pearson correlation coefficient/ Spearman rank correlation coefficient ≥ 0.80; “−” ICC < 0.70 or Pearson correlation coefficient/Spearman rank correlation coefficient < 0.80;

Content validity: “+” The content of the assessment instrument is adequate, comprehensive, questions and tasks chosen to adequately reflect the content to be evaluated; “−” Not all selected questions and tasks reflect the content, content is not relevant, comprehensive;

Criterion validity: “+” Significant and stable relation between measurement and another instrument (r ≥ 0.70) or with start/end measurement; “−” Poor measurement correlation with another instrument (r < 0.70) or start / end measurement;

Structural validity: “+” Correlation with instruments measuring the same ≥ 0.50 or correlation higher for unrelated elements in the instrument than for unrelated ones; “−” Correlation with instruments measuring the same < 0.50 or correlation with related elements in the instrument is lower than unrelated ones;

Responsiveness: “+” Able to detect clinically significant changes over time; “−” Cannot detect clinically significant changes over time

^*^Except Cognitive scale

## Discussion

This study identified nine rehabilitation assessment instruments that have most commonly been referred to in the literature for adults with brain tumors, that cover all components of the ICF, and have good psychometric properties. As far as the authors are aware, this is the only scoping review of assessment instruments used for adults with brain tumors. However, this scoping review did not identify one unique assessment instrument for the target group. This patient group is specific in a way that there is no unifying patient-specific clinical set of symptoms and their symptoms depend on various other factors [14].

Five of these tools are used for objective assessment: KPS, MMSE, FIM, TMT, BI, four are self-assessment tools: FACT-Br, SF-36, EORTC QLQ-C30, EORTC QLQ- BN20. One of these tools (KPS) is used to assess physical activity, two (MMSE; TMT) are cognitive function assessment tools, FACT-Br, SF-36, EORTC QLQ-C30, EORTC QLQ-BN20 measures the quality of life, and both, FIM and BI are used to assess disability.

The most frequently used assessment instrument is the Karnofsky Performance Scale as it is used as a criterion for the selection of participants by measuring their level of physical activity [15]. This assessment tool is developed for a general assessment of oncological patients [16] and reflects the overall ability to perform usual daily activities (component of Activities and Participation of the ICF) in the context of help needed from other people (Environmental Factors).

Four of these instruments are used to evaluate patients with brain tumors: EORTC QLQ-C30; EORTC QLQ-BN20; FACT-Br, SF-36; they are all linked to the quality of life. Moreover, the EORTC team recommends that EORTC QLQ-C30 and EORTC QLQ-BN20 tools be used together [17]. These two tools cover both functioning components of the ICF and from the perspective of content, complement each other. EORTC QLQ-C30 contains more specific questions on problems specific to patients with brain tumors [18, 19]. The EORTC QLQ-C30 and the EORTC QLQ-BN20 provide comprehensive information about the patient's quality of life, but this is often overlooked in studies identified in this scoping review. The FACT-Br questionnaire has been used less and it has as good properties in terms of intra-rater reliability and structural validity as other two specific quality of life measurements, contains problems that have not been included in any of the previous tools, and can clearly be important for this population, such as handling stress or driving a car. It also considers important Environmental factors, such as help and attitudes of family members and friends, as well as health professionals. Some important concepts also overlap with the SF-36 that have developed as a multipurpose tool that is used for assessment of functional health and well-being [20] and has also been widely used for patient-reported outcomes in populations with different diagnoses [21]. Therefore, this could be a good choice to use the SF-36, if the comparison between populations is needed.

Between the most used assessment tools, the FIM and BI have been listed. These instruments are non-specific to diagnosis, and both have been widely used in different rehabilitation populations [22–24]. Both scales, the FIM and the BI, are performance-based assessment tools and both analyze the level of independence in the most important activities of daily living. Their psychometric properties have been profoundly analyzed, and the ceiling effect for the BI can be observed when compared to the FIM [25]. However, the psychometric properties of the objective assessment instruments specific for the patient group have not been proven; therefore, their psychometric properties were demonstrated in patients suffering from stroke, traumatic brain injury (TBI), or similar neurological conditions. Interestingly, two neuropsychological assessment tools (the MMSE and the TMT) are mentioned among the most frequently used for persons with brain tumors. It can be explained by the fact that cognitive impairments are a common symptom in patients with brain tumors [4]. Both instruments focus mostly on the cognitive functions of the component of the Body Functions and Structures of the ICF and both are performance-based. However, the psychometric properties of the MMSE have been better documented.

The International Classification of Functioning, Disability and Health (ICF) provides the user with a broad spectrum of health outcomes, including physical and cognitive functioning. By linking available assessment tools to this concept, it is possible to analyze the content of the available instruments and choose the appropriate one for the problem that is being measured and, consequently, treated [11, 12]. Using the ICF framework, it was possible to link most elements identified in the assessment instruments to certain categories. Some elements could not be linked since they covered topics such as quality of life, personal factors, or certain elements not defined in the ICF. Body Function categories were dominated by MMSE, TMT, EORTC QLQ-BN20, EORTC QLQ-C30, activity and participation categories—FIM, BI, KPS, SF-36 but FACT-Br viewed these two domains equally. Environmental factors were assessed by EORTC QLQ-30, FACT-Br, FIM, BI, and KPS. Given that the clinical picture of brain tumor patients is similar to that of other neurological conditions, such as stroke [6] or TBI [7], the ICF Core Sets were reviewed for stroke and TBI [26], and their categories were compared to categories identified in this scoping review. Comprehensive core sets for stroke listed 13 categories in body functions and structures, 14 in activities and participation, and 23 categories in environmental factors that were not identified in assessment instruments analyzed in this study. Comprehensive core sets for TBI listed 10 categories in body functions domain, 22 in activities and participation, and 28 categories in environmental factor domain that were not identified in any of 9 assessment instruments analyzed within this study. This can be explained by the fact that the most frequently used assessment instruments do not cover all the possible impairments for people with brain tumors.

Given that the ICF Core Sets for stroke and TBI were compared to categories identified in this review and they proved to be overall covering similar areas it can be concluded that all 9 assessment tools identified in this study can be appropriate and specific assessment instruments for patients with brain tumors, as they have been proven valid, reliable, and responsive to a variety of neurological conditions. Further research is recommended to assess reliability, validity, and responsiveness of assessment instruments specifically for brain tumor patient groups.

Overall, the current study has a few limitations. First, the quality of the studies included in the scoping review was not assessed, as the purpose of the scoping review was to identify the most frequently used assessment instruments. Second, the authors included only nine out of 86 assessment tools for further analysis, which were used in more than 9% of the study articles included in the study. That runs the risk that this analysis of assessment instruments does not use some of the more recently developed assessment tools, which may be better suited for the specific patient group but are not used frequently enough in research articles to be included in the analysis.

## Conclusions

Between the nine most frequently used assessment instruments in clinical studies, one was a generic tool for an overall description of activity level for patients with diagnosis of cancer, three were diagnosis-specific self-assessment tools, one was a multipurpose tool for assessment of functionality and health status, two were widely used tools in rehabilitation for assessment of activities of daily living, and two were neurocognitive tests. These tools cover all components of the International Classification of Functioning, Disability and Health and have proven to have good psychometric properties; however, the assessment tools that are not diagnosis-specific, still must be validated for the brain tumor population.

Since the content and administration vary, the choice of the tool used for assessment of patients with brain tumor depends on the clinical question posed, as well as the aim of the use of this tool.

## Supplementary Information

**Additional file 1**. All identified assessment tools, frequency and references. 

## Data Availability

The datasets used and/or analysed during the current study are available from the corresponding author on reasonable request.
